# Manipulation of Type I Interferon Signaling by HIV and AIDS-Associated Viruses

**DOI:** 10.1155/2019/8685312

**Published:** 2019-04-04

**Authors:** Buyuan He, James T. Tran, David Jesse Sanchez

**Affiliations:** Pharmaceutical Sciences Department, College of Pharmacy, Western University of Health Sciences, Pomona 91766, California, USA

## Abstract

Type I Interferons were first described for their profound antiviral abilities in cell culture and animal models, and later, they were translated into potent antiviral therapeutics. However, as additional studies into the function of Type I Interferons progressed, it was also seen that pathogenic viruses have coevolved to encode potent mechanisms allowing them to evade or suppress the impact of Type I Interferons on their replication. For chronic viral infections, such as HIV and many of the AIDS-associated viruses, including HTLV, HCV, KSHV, and EBV, the clinical efficacy of Type I Interferons is limited by these mechanisms. Here, we review some of the ways that HIV and AIDS-associated viruses thrive in Type I Interferon-rich environments via mechanisms that block the function of this important antiviral cytokine. Overall, a better understanding of these mechanisms creates avenues to better understand the innate immune response to these viruses as well as plan the development of antivirals that would allow the natural antiviral effect of Type I Interferons to manifest during these infections.

## 1. Introduction

Type I Interferons (IFN) were first described for their ability to interfere with viral infection in the 1950s [1, 2]. The ability of many types of cells to induce IFN in response to viral infection, and the subsequent ability for IFN to stimulate a block to viral infection in many cell types, has given IFN an exciting role as an all-encompassing antiviral therapeutic. However, almost seventy years of research have shown that with all its power as an antiviral therapeutic, the pathogenicity of almost all viruses seems to require the encoding of countermeasures that subvert the IFN response. In particular, chronic viral infections seem to thrive in microenvironments that produce relatively high levels of IFN, yet these viruses still persist. Here, we focus on recent advances in understanding the subversion of IFN signaling during HIV infection and AIDS, as well as how several other chronic viruses continue their replication in the face of a robust IFN response.

## 2. IFN and Clinical HIV Infection

The most effective regimen to treat against HIV is highly active antiretroviral treatment (HAART) with physicians prescribing these combination antiretroviral therapies (cART) to limit the replication of HIV down to undetectable levels. IFN itself is a potent antiviral treatment that can activate the antiviral state in the host during HIV infection. Nevertheless, there had been many controversies on whether IFN is an effective treatment for HIV due to its positive effect on acute viral infection and negative effect on chronic infection. Cheng et al. showed that cART inhibited HIV replication but failed to completely stop elevated ISG expression, implying a sustained IFN induction [3]. Sustained IFN is believed to be partially responsible for immunological exhaustion that could lead to diminished T-cell function in chronic HIV infection [3–5]. To tackle this problem, Cheng et al. developed a monoclonal antibody to block IFN-*α*/*β* receptor (IFNAR) in humanized mice infected with HIV-1. The anti-IFNAR1 mAb suppressed ISG expression in humanized mice with a functional human immune system (hu-mice) and HIV-infected hu-mice, and it subsequently rescued anti-HIV-1 T-cell function. Importantly, the decrease in ISG levels that was seen with anti-IFNAR mAb therapy led to a decrease in viral load. This was suggested to be due to a decrease in PD-1^+^, a suppressive CD8^+^ T-cell which is normally important for suppressing overactive immunity. An additional finding focused on the fact that if these anti-IFNAR mAbs are administered during cART therapy, HIV-1 rebound after cART was delayed in the anti-IFNAR mAb-treated animals. This strategy can provide a novel therapeutic approach to treat patients living with HIV-1 infection with a sustained IFN-I level during cART [3]. In another study, elevated IFN-I signaling during chronic HIV infection was shown to be the main cause for underlying chronic inflammation, immune activation, and CD8^+^ T-cell exhaustion [6]. Here, they showed that the combination of ART and IFNAR blockade during chronic HIV infection increased viral inhibition and ultimately led to a reduction in the reactivatable HIV reservoir. The study highlights the importance of IFN during viral infection and supports the ideas that IFN may act on both sides of the table during chronic HIV infection, fueling persistent immune activation and viral dissemination [6].

HIV-specific antibodies (Abs) can also have an effect on IFN that is produced by plasmacytoid dendritic cells (pDCs). Monoclonal Abs that interact with HIV gp120-CD4 binding suppress the IFN response, suggesting that gp120-CD4 interaction is critical for IFN production by pDCs [7]. As a result, vaccination or other treatments that can interrupt HIV gp120 at the CD4 binding site relative to the binding of other HIV envelope epitopes may have therapeutic potential in reducing immune activation beyond just the neutralization of the virus. This finding also suggests that the selection of mAb, based on the pDC production of IFN, should be considered carefully for clinical trials because they could lead to an increase in immune activation as mAb that did not block gp120-CD4 binding could lead to increased IFN responses [7].

In contrast to the negative effect of IFN during chronic HIV infection, stimulation by IFN is necessary to inhibit HIV spreading during acute infection. The production of IFN is stimulated by many pattern-recognition receptors that sense HIV-1. Activated CD4^+^ T-cells recognize HIV-1 infection through cytosolic DNA sensor cGAS and induce IFN response. This response is regulated by two viral accessory proteins in CD4^+^ T-cells [8]: Vpr, as it increases HIV-1 sensing, and Vpu, as it suppresses cGAS-dependent IFN induction. In other cases of HIV infection, T-cells were shown to have a defect in DNA signaling machinery, which results in DNA sensing that does not lead to the activation of innate response. The subsequent lack of expression of ISGs, IFN, and proinflammatory cytokine leads to a failure to induce an antiviral state that is sufficient to suppress HIV spread from infected cells. The data pose a question forward as to why DNA-sensing machinery is defective in T-cells but functional in other cell types [9].

Of all the interferon subtypes, IFN-*α*2a had been tested in many clinical trials to test its safety and effectiveness. One clinical trial of eleven volunteers living with HIV infection underwent 12 weeks of therapy with pegylated interferon alfa-2a [10]. The median plasma viral load reduction and CD4^+^ T-cell counts at week 12 were 0.61 log_10_ copies/mL and -44 cells/*μ*L, respectively. This showed that IFN-*α*2a was tolerated, and it portrayed a significant anti-HIV-1 activity in HIV-1-infected patients [10]. In another clinical trial with 9 participants living with HIV-1 infection that were also treated with pegylated interferon alfa-2a [11], a subset of ISGs (23 of 47) increased compared to the baseline by week 6, while 10 ISGs were inversely correlated with responses to the virus. The results indicated that the HIV virologic response by pegylated interferon alfa-2a only includes a specific ISG subset [11]. In one other clinical test, viral suppression was detected in 9 out of 20 patients who received pegylated interferon alfa-2a monotherapy for 12 weeks [12]. Patients who had a viral load of <400 copies/mL had reduced levels of integrated HIV DNA compared to those who experienced end-point failure. The data was further supported by the control of HIV replication and the reduction of HIV-1 integration due to pegylated interferon alfa-2a monotherapy [12]. Although IFN has been used to treat HIV, it is also crucial to consider which specific subtypes of IFN should be used to treat different patients with different viral infections. One study demonstrated the increased potent anti-HIV response of the human IFN-*α*14 subtype in humanized mice compared to IFN-*α*2 [13]. The finding suggests that although IFN-*α*2 is currently approved as a therapy of HBV and HCV, it is critical to determine if IFN-*α*2 is the most effective and safe subtype for other viruses [13].

## 3. HIV Disrupts IFN Induction during Treatment

**Figure 1:**
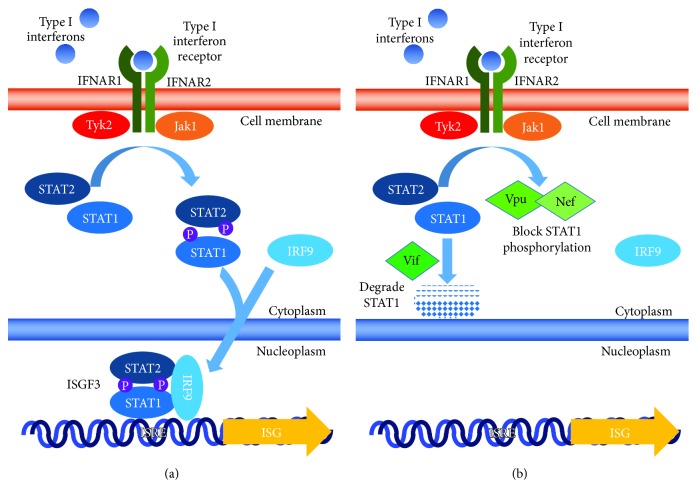
Interferon induction in normal and HIV-infected cells. (a) Interferon induces the induction of interferon-stimulated genes (ISG) through the activation of ISGF3. (b) HIV Vif leads to the degradation of STAT1 and Vpu, and Nef blocks phosphorylation of STAT1.

Why are there so many inconsistencies when dissecting the efficacy of IFN during therapy for HIV-1 infection? Although IFN-I treatment is normally essential for viral clearance, HIV-1 has evolved several mechanisms to bypass or suppress the effect of IFN in multiple situations. Numerous studies have been conducted to determine the mechanisms that HIV utilizes to reduce the effectiveness of both endogenous and therapeutic IFN-*α*, which leads to less control over HIV infection. HIV is able to use many of its accessory proteins to interrupt different mechanisms to suppress and evade the host immune system. The protein Vif of HIV has been proposed to play a role in its own catalysis, in the ubiquitination and proteasomal degradation of STAT1 and STAT3 proteins of the JAK/STAT pathway, and in the degradation of monocytic cell lines, which allows HIV-1 to block the antiviral effects of IFN-I. More specifically, Vif-mediated STAT1 and STAT3 inhibition reduces IFN-*α* induction of ISG-15 [14]. On the other hand, another work has shown that Vpu and Nef are able to block the phosphorylation of Nef without the degradation of STAT1 in T-cell lines [15]. These studies show that multiple mechanisms, which may be cell-type specific, may explain the therapeutic failure of interferon on HIV-1 infection (summarized in Figure 1).

Additionally, others have shown that there is a decrease in the gene expression level of IFN-*λ*1, IFN-*β*, and RANTES in HIV-1 patients after primary cells are transfected with foreign DNA compared to cells from uninfected patients. This implies that in patients who live with HIV-1 and have undetectable (<50 copies/mL) viral loads, there are lower innate responses through the cytosolic DNA-sensing system. This attenuation of innate immune responses may be due to persistent immune activation [16].

## 4. HIV Blocks IFN Induction in Many Cell Types

HIV-1 also is able to block type I and III IFN induction in human dendritic cells and macrophages. To do this, HIV-1 specifically inhibits the phosphorylation of TANK-binding kinase 1 (TBK1). Deletion of Vpr and Vif, two HIV-1-encoded proteins from the HIV-1 genome, leads to detectable IFN-I induction. Vpr and Vif were shown to bind to TBK1 and disrupt the process of TBK1 transautophosphorylation, subsequent IRF3 phosphorylation, nuclear translocation, and induction of IFN-I and IFN-III gene expression [17]. Other groups have shown that Vpu and Nef proteins from HIV-1 lead to the degradation of IPS-1, an essential adaptor protein in the innate immune recognition of viral RNA by the RIG-I-like receptor family (Sanchez, 2015). In that study, deletion of Vpu and Nef from the HIV-1 genome leads to an HIV-1 infection that could not degrade IPS-1 and could induce IFN release.

Vpu proteins of the HIV-1 group M strains have been demonstrated to disrupt the restriction factor tetherin, which suppresses virus release from infected cells. A study introducing the mutation of *vpu* genes from HIV-1 group M strains, the predominant strains of HIV-1, and N strains, an uncommon strain of HIV-1, showed that they were able to interrupt their function to antagonize tetherin. This decreased the ability of the Vpu protein to antagonize IFN-mediated virus restriction and resulted in less virus production and release from CD4^+^ T-cells, from fivefold to twofold, with higher levels of IFN-I released. This suggests the essential role of the Vpu protein in counteracting the human tetherin during viral infection and controlling IFN release [18].

## 5. HIV Disruption to pDC-Induced IFN

The majority of IFN released during viral infection is produced by plasmacytoid dendritic cells (pDCs). Other viruses, such as influenza or HSV, induce IFN-*α* production by pDCs within 4 hours to maximal levels. On the other hand, IFN-*α* induction was delayed by 24 hours by HIV infection, and the maximal level was at least 10-fold less than other viruses. Looking closer, SYK phosphorylation at numerous tyrosine sites was observed after the exposure to HIV and gp120. This indicated that HIV may hijack the BDCA-2 signaling pathway, which then leads to the inhibition of IFN production in pDCs [19]. Gp120, an HIV-1 envelope protein, also plays an essential role in the inhibition of IFN-*α* secretion in pDCs. Gp120 was observed to interact with Toll-like receptor 9 (TLR9) in pDCs and subsequently obstruct the induction of IFN-*α*. Furthermore, natural killer (NK) cells that were activated by pDCs to kill target cells were found to portray decreased cytolytic activity after TLR9 agonist- (CpG) treated pDCs were exposed to gp120 [20].

## 6. HIV Targets IFN-Induced ISGs

Another mechanism that HIV utilizes to avoid IFN therapy is to downregulate a number of IFN-stimulated genes (ISGs). HIV was found to suppress multiple ISGs, including AXL, OAS1, and XAF1, with a fold change greater than 1.5. This phenomenon demonstrates how the virus is still able to downregulate many antiviral ISG transcriptions despite the fact that the virus replication is suppressed by IFN pretreatment [21]. The infection of HIV on T-cells and macrophages often does not trigger the innate immune system to produce IFN. One component that assists HIV to evade the innate immune system is the cytoplasmic exonuclease TREX1. Data from both macrophages and CD4^+^ T-cells show that HIV infection leads to IFN production when TREX1 is suppressed by RNA interference (RNAi). This suggests that TREX1 interacts with and digests excess cytosolic HIV DNA that would generally stimulate IFN expression. The results also demonstrated that the signaling cascade through STING, TBK1, and IRF3 to induce IFN expression is blocked [22].

More interestingly, in another scenario, HIV is able to use IFN-I to the virus's advantage to further damage the host immune system. B cell-activating factor (BAFF) expression and secretion have been observed to be upregulated in human monocytes which were induced by HIV-1. More specifically, HIV-1 has been shown to induce IFN production by plasmacytoid dendritic cells (pDCs), which result in increased production of BAFF. The high expression of BAFF often leads to B cell dysfunctions, including hypergammaglobulinemia and nonspecific B cell activation. These findings highlight a mechanism for the enhanced BAFF levels during HIV-1 infection and the importance of pDC and monocyte crosstalk to stimulate BAFF secretion [23].

## 7. HTLV and IFN-1

The modulation of IFN by other viruses is another facet of virus pathogenicity especially in people living with HIV infection or with full-blown AIDS. The human T-lymphotropic viruses (HTLV), types I and II, are another class of retroviruses that affect T-cells. Usually, there are no signs or symptoms that can be observed, but some affected people may develop adult T-cell leukemia- (ATL-) and HTLV-1-associated myelopathy/tropical spastic paraparesis (HAM/TSP). Many types of treatment, including IFN, were tested to understand the pathology of this virus and to determine the right therapy for patients. HTLV-1 encounters different types of dendritic cells (DCs) that are in blood, intestinal, and genital mucosa during blood or sexual transmission. These differences can alter HTLV-1's ability to infect DCs and transfer to T-cells. A few studies emphasized the idea that DCs are more susceptible to infection by HTLV-1than other cell types. A higher proviral load was observed in monocyte-derived dendritic cells (MDDCs) compared to lymphocytes that were exposed to a viral biofilm. The expression of neuropilin-1 observed in MDDCs was also higher than that observed in activated T lymphocytes. Furthermore, MDDCs could transfer virus to lymphocytes efficiently [24]. Another study had similar results as they showed that DCs exposed to HTLV-1 can efficiently induce the transmission of the virus to autologous primary CD4^+^ T-cells. Neuropilin-1 is involved in the process of DC-mediated transfer of HTLV-1 that leads to the efficient infection of CD4^+^ T-cells [25]. The susceptibility of DCs to HTLV-1 infection was further examined to understand the mechanism of viral interaction with DCs. DC-specific intercellular adhesion molecule-3-grabbing nonintegrin (DC-SIGN) was found to be a critical DC antigen receptor. DC-SIGN was shown to mediate HTLV-1 transmission from DCs to T-cells. The increase in virus-induced interleukin-4 production and DC-SIGN expression leads to the successful HTLV-1 infection of MDDCs in blood myeloid DCs. These data reveal the essential role of DC-SIGN in HTLV-1 infection and transmission and provide a potential target for antiviral therapy development [26]. A study demonstrated that IFN-*α*-stimulated DCs significantly restrict HTLV-1 infection more than monocyte-derived IL-4-stimulated DCs and TGF-*β*-stimulated DCs despite their enhanced ability to capture HTLV-1 virions. This was not because of IFN antiviral activity, but this was related to the distinct trafficking route of HTLV-1 in IFN-*α*-stimulated DCs compared to other DCs [27]. As IFN is one of the important effectors of the innate immune response, IFN was reported by multiple groups to work in a variety of ways with different mechanisms that contribute to the inhibition of HTLV-1. HTLV-1 mRNA and proteins in HTLV-1-infected cells were demonstrated to be reduced when cocultured with human epithelial-like cells (HEK293T) or mouse embryo fibroblasts (NIH 3T3). The positive effect from these cocultures was due to IFN induction, which induced IFN-beta promoter activation and ISG upregulation. Furthermore, the suppression of HTLV-1, mediated by HEK293T and NIH 3T3, can also be inhibited by antibodies to the human IFN-*α*/*β* receptors or mouse IFN-*β*. The results suggest that the innate immune response may inhibit HTLV-1 expression *in vivo* through IFN [28]. Recently, the combination of IFN-*α* and zidovudine (AZT) was shown to have the most therapeutic effects in adult T-cell leukemia/lymphoma (ATL) patients compared to other treatments. One study found the level of HTLV-1 p19, a major core viral protein encoded by the HTLV-1 *gag* gene, in the supernatant of IL-2-dependent HTLV-1-infected T-cells (ILTs) reduced within three days after IFN-*α* stimulation. Moreover, the amount of intracellular Tax viral proteins, in 6 of 7 ILT lines tested, was observed to decrease after 24 hours following IFN-*α* stimulation. The treatment of AZT alone did not influence HTLV-1 gene expression, NF-*κ*B activities, or cell viability. However, the treatment of AZT and IFN-*α* generated p53 signaling and promoted cell apoptosis in these cells. These data suggest that the susceptibility of HTVL-1-infected cells to IFN-I response is at the IL-2-dependent stage, and partially reveal the therapeutic effects of AZT/IFN-*α* in ATL [29]. A clinical study measured the activity of HTLV-1 reverse transcriptase (RT) and other viral components, by quantitative real-time PCR, in samples from cultures of peripheral blood mononuclear cells (PBMCs) that were collected from 7 ATL patients before and after AZT and IFN treatment. HTVL-1 *tax*/*rex* expression in PBMC cultures from 4 patients was variably inhibited compared to pretreatment samples. Analysis of p19 production showed a decrease from 75% to 88% when supernatant from PBMC cultures of 5 patients was measured. And most importantly, RT activity was significantly suppressed in samples collected from 5 patients after AZT/IFN therapy [30].

Nevertheless, multiple researchers have reported that HTLV-1 utilizes many different viral proteins, with different mechanisms, to regulate IFN response. In Jurkat or HEK293 cells, Tax was observed to interrupt TBK1 kinase that phosphorylates IRF3 that leads to the inhibition of IFN production [31]. Furthermore, Tax was shown to be recruited to cellular immunocomplexes with TBK1 and I kappa B kinase (IKK*ɛ*) that normally lead to the phosphorylation of interferon regulatory factors that stimulate *IFN* expression. *IFN-β* promoter activity was increased with the expression of Tax in the presence of TBK1 and IKK*ɛ*. A mechanism is proposed in [32] wherein Tax is recruited as a scaffold protein between IFN-*β* signaling factors and the kinase complexes, allowing TRAF3 to interact with the TBK1/IKK*ɛ* complex and activate the *IFN-β* promoter. HTLV-1 bZIP factor (HBZ), another HTLV-1-encoded protein, also plays an essential role in viral pathogenesis. HBZ was shown to upregulate IRF7-induced ISRE (IFN-stimulated response element) promoter activities and IFN-*α* that could offset the inhibitory effect of Tax1 on IFN-*α*. On the other hand, the combination of HBZ and Tax1 synergistically impedes IFN-*β* and ISRE promoter induction that would lead to IFN-*β* production. Furthermore, HBZ was demonstrated to regulate, positively or negatively, TBK1 and IKK*ε* activation of IRF7 and IRF3. These results suggest that the variety of regulation is orchestrated by HTLV-1 on IFN response and may contribute to aberrant IFN signaling, immune evasion, and viral pathogenesis [33]. All these data present different views on whether IFN is efficient enough to treat HTLV-1 and if HTLV-1 is able to bypass the effect of IFN-I therapy, either alone or in combination with other components. Further research on HTLV is necessary to comprehend the mechanisms behind the role of both IFN and HTLV-1 viral proteins in order to produce the most sufficient and effective therapy for HTLV-1.

## 8. HCV and IFN-1

Hepatitis C virus (HCV) is a single-stranded RNA virus belonging to *Flaviviridae* and infects about 3.9 million people in the United States, establishing chronic infection in about 2.7 million people. Persistent infection of HCV can be the cause of chronic hepatitis, liver cirrhosis, and hepatocellular carcinoma [34]. IFN therapy was once key in the treatment of Hepatitis C with a decrease of HCV RNA levels in serum being detected [35]. However, therapy with only IFN has limited success and here we discuss how HCV applies mechanisms to counter IFN treatment. It should be noted that the advent of direct acting antivirals (DAAs) for HCV has moved most therapeutic regimes away from IFN therapy [36, 37].

Several HCV proteins have been shown to inhibit IFN signaling. HCV encodes a nonstructural-protein 5A (NS5A) that has been shown to disrupt the function of several ISGs [38]. PKR is a well-studied ISG, with the activation of PKR leading to the phosphorylation of eukaryotic initiation factor 2*α* (eIF2*α*) and a subsequent block to the translation of viral mRNAs [39]. Gale et al. demonstrated that the HCV NS5A protein could downregulate PKR by directly interacting with its protein kinase catalytic domain and could thus repress PKR functions [40]. They also found that an interferon sensitivity-determining region (ISDR), as well as the addition of a 26-amino-acid carboxyl to the ISDR, is needed for NS5A/PKR interaction [41]. Meanwhile, Sugiyama et al. used a recombined JFH-1 virus, a recombinant HCV derived from a genotype 2a isolate of HCV, that had the HCV ISDR and proved the essential role of the ISDR to inhibit IFN signaling [42]. Since the proinflammatory chemokine IL-8 has been shown to interfere with the IFN-mediated pathways such as by reducing 2′-5′oligoadenylate synthase (OAS) activities [43], the ability of NS5A to induce IL-8 production could be another mechanism inhibiting the antiviral activities of IFN [44]. The upregulation of IL-8 by NS5A was also shown by Girard et al. using microarray assays [45].

Other HCV proteins have also been shown to inhibit PKR activity. Since PKR phosphorylates eIF2*α*, the HCV envelope 2 (E2) protein contains a sequence homologous to the eIF2*α* phosphorylation site. Taylor et al. performed an in vitro binding assay and proved E2's binding to PKR, which could be the mechanism of E2 inhibiting PKR activities [46].

IFN activates the transcription of ISGs through the JAK/STAT pathway [47], and the HCV core has been widely demonstrated to modulate the JAK/STAT pathway. Hosui et al. found that in CL2 cells expressing the HCV core protein, the phosphorylation of JAK1, JAK2, and STAT3 were all lower compared with mock CL2 cells, while the expression of the HCV core protein did not significantly affect the expression level of those proteins [48]. Lin et al. described the ability of HCV to inhibit IFN signaling by degrading STAT1 and inhibiting its phosphorylation [49]. Also, they explained this mechanism as they discovered that the N-terminal of the HCV core protein interacts with the STAT1 SH2 domain [50].

Furthermore, the HCV core protein is reported to upregulate miR-93-5p, which blocks IFN signaling by directly targeting the interferon receptor IFNAR1 [51]. Besides miR-93-5p, other microRNAs also regulate IFN signaling. Since miR-373 expression is induced by HCV infection, Mukherjee et al. found that miR-373 expression inhibits JAK and IRF9 while also blocking STAT1 phosphorylation. On the other hand, knockdown of miR-373 induces an enhancement of IFN signaling proteins and a reduction of HCV growth [52].

The activation of the Ras/Raf/MEK pathway is reported to be involved in a large proportion of cancers [53]. Zhang et al. addressed the correlation between the Ras/Raf/MEK pathway and HCV infection. HCV infection is found to activate this pathway, and this activation of the pathway blocks the expression of IFNAR1/2 and the phosphorylation of STAT1/2, thus inhibiting the JAK/STAT pathway which would induce ISG [54].

Additionally, other factors also affect HCV's inhibition of IFN signaling. Recently, the extracellular matrix (ECM) has been shown to affect IFN signaling in HCV-infected cells. Kuwashiro et al. compared HCV-infected human hepatoma cells cultured on ECM-coated dishes or noncoated dishes. In cells grown on ECM-coated dishes, ISRE luciferase activities were lower, while HCV-RNA and viral protein amounts were higher. Also, antibodies blocking the cell-matrix interactions were able to restore the ISRE luciferase and reduce viral RNA/protein amounts, showing ECM's role in IFN signaling [55].

## 9. KSHV and IFN-1

Kaposi's Sarcoma-associated Herpesvirus (KSHV), also known as Human Herpesvirus 8, was identified in Kaposi's Sarcoma (KS) lesions during the AIDS epidemic. KSHV was later shown to cause lymphoproliferative disorders including primary effusion lymphoma (PEL) diseases and multicentric Castleman's disease (MCD) [56].

The KSHV genome encodes a group of genes homologous to human interferon regulatory factors (IRFs), including vIRF1 (viral IRF1), vIRF2, and vIRF3 which have been shown to have a different impact on blocking IFN genes or ISGs [57]. Several studies reported that vIRF1 is able to disrupt IFN signaling by blocking the ISG promoters, including ISG-15 and ISG-54 [58]. vIRF3 was previously shown to interact with IRF3, IRF5, and IRF7. For example, Wies et al. described the ability of vIRF3 to interact with IRF5 and inhibit ISG transcription by impacting ISRE elements [59]. vIRF3 was also found to inhibit the PKR-activated phosphorylation of eIF2*α* and PKR-induced inhibition of protein synthesis, thus impairing the antiviral ability of PKR [60].

vIRF2, encoded by ORF K11.1, is able to interfere with multiple sites of IFN signaling. Through the ISRE luciferase assay, Fuld et al. showed that the full length of vIRF2 inhibits ISRE signaling induced by IFN-*α*, IL-28A, IL-29, and also IRF1 [61]. During IFN signaling, STAT1 and STAT2 bind to the ISRE with IRF9 and form a complex called ISGF3, which facilitate transcription of ISGs. vIRF2 was shown to inhibit the ISGF3 complex by targeting STAT1 and IRF9, both key components of the ISGF3 complex [62]. Additionally, vIRF2 also interacts with PKR and blocks its autophosphorylation or phosphorylation of eIF2*α* [63].

KSHV viral IL-6 is another well-known KSHV viral homologous gene that has been described to block the phosphorylation of Tyk2 which leads to an inhibition of the formation of the ISGF3 complex [64]. Notably, KSHV RIF (Regulator of IFN Function), encoded by KSHV ORF10, is able to attenuate IFN signaling by a similar mechanism. RIF is found to inhibit downstream signaling of IFNAR by associating with JAK1, STAT2, and Tyk2. Besides, RIF is also shown to interact with both IFNAR1 and IFNAR2 units, forming an inhibitory complex. Therefore, RIF blocks the phosphorylation of STAT1 and STAT2, impairing the form of the ISGF3 complex [65].

During latency, KSHV encodes 12 premicroRNAs, which are processed to at least 25 miRNAs [66, 67]. KSHV miRNAs including miR-K6-5, miR-K8, and miR-K9 are found to downregulate STAT3 phosphorylation [67–69]. KSHV miRNAs are also shown to inhibit several targets associated in the STAT3 signaling network, including upstream components, such as IRAK1, PKC*δ*, EPOR, and MET, or downstream components like BIRC5 and GADD45B [67]. In addition, another study showed that miR-17 could also target Jak1, downregulating its mRNA level and subsequent protein level, thus impairing IFN signaling [70].

## 10. Epstein-Barr Virus and IFN-1

Epstein-Barr virus (EBV) is another species of *γ*-herpesvirus, which is reported to be carried by 90% of the human adults asymptomatically and persistently [71]. In order to persist in a host body for the entire life of the host, EBV encodes certain strategies to escape from immune detection, and IFN in particular, as needed.

Latent membrane protein-1 (LMP-1) is a latent oncoprotein essential for EBV persistency in B cells [72], which shares many signaling intermediates with TLRs and also activates NF-*κ*B [73]. Geiger and Martin demonstrated LMP-1's ability to interact with Tyk2 and inhibit the phosphorylation of Tyk2 and STAT2, thus blocking the activation of ISREs. Also, higher levels of LMP-1 are observed in EBV-infected lymphoblastoid cells cultured in IFN, suggesting LMP-1's function in resisting antiproliferative pressure [74]. However, other studies also showed LMP-1's ability to induce STAT1 expression by its C-terminal-activating region 1 (CTAR-1) [75]. Moreover, the C-terminal-activating regions of LMP-1 are also reported to induce IFN [76]. This contradiction may explain the multiple roles of LMP-1 in maintaining cell survival but also inhibiting immune responses that threaten the latent virus.

Latent membrane protein-2 (LMP-2) is designated as LMP2A and LMP2B. LMP2A, being a viral mimic of the B-cell receptor, has been described to promote viral latency and cell survival [37, 77]. In addition, in EBV-infected endothelial cells, LMP2A is also shown to inhibit both STAT signaling and NF-*κ*B signaling. Previously, it has been reported that LMP2B is a negative modulator of LMP2A activities [78]. However, LMP2B is also shown to cooperate together with LMP1A to inhibit IFN signaling. Both LMP2A and LMP2B are found to inhibit IFN-induced ISRE activity by blocking JAK/STAT1 phosphorylation. Consequently, they attenuate ISG transcription, which is found “globally” [79].

After a screening of EBV open reading frames by Wu et al., the tegument protein LF2 is found to specifically inhibit ISRE activation induced by cellular IRF7 but not that induced by IRF3. Moreover, LF2 does not affect the IRF7 level but blocks the IFN signaling by binding to the central inhibitory association domain of IRF7, which causes an inhibition of the dimerization of IRF7 [80].

EBV encodes two nonpolyadenylated RNAs (EBERs), among which EBER1 is found to disrupt the antiviral effects of IFN-*α* and IFN-*γ* by interacting with PKR and blocking its function [81]. EBV has also been shown to encode more than 40 microRNAs [82]. Within a cluster of 10 EBV BART miRNAs impairing IFN responses, miR-BART16 is identified to be the major inhibitor of the IFN-induced ISRE activity, while other miRNAs also contribute to this repressive impact. Furthermore, CBP (CREB-binding protein) is identified to be the target of miR-BART16, which consequently attenuates the antiproliferative effect of IFN-*α* [83].

Suppressor of cytokine signaling (SOCS) is a family of cellular proteins that inhibits cytokine signaling pathways, inhibiting IFN signaling by negative feedbacks [84]. Besides EBV viral proteins and RNAs, EBV infection also induces the activation of SOCS3, which suppresses IFN signaling by blocking the JAK/STAT pathway [85].

## 11. Conclusion

While IFN was discovered for its powerful antiviral impact on innate immunity, the large numbers of anti-IFN strategies that are encoded in so many viruses underscore the coevolution that viruses have undertaken with humans and other hosts. With our understanding of viral pathogenesis constantly growing, it is now an opportune time to focus on developing strategies to open up the antiviral potential of IFN by targeting the many ways that viruses have developed to avoid IFN.

## References

[1] Isaacs A., Lindenmann J. (1987). Virus interference. I. The interferon. *Proceedings of the Royal Society of London - Series B: Biological Sciences*.

[2] Isaacs A., Lindenmann J., Valentine R. C. (1957). Virus interference. II. Some properties of interferon. *Proceedings of the Royal Society of London - Series B: Biological Sciences*.

[3] Cheng L., Ma J., Li J. (2017). Blocking type I interferon signaling enhances T cell recovery and reduces HIV-1 reservoirs. *The Journal of Clinical Investigation*.

[4] Seung E., Dudek T. E., Allen T. M., Freeman G. J., Luster A. D., Tager A. M. (2013). PD-1 blockade in chronically HIV-1-infected humanized mice suppresses viral loads. *PLoS One*.

[5] Zhang L., Su L. (2012). HIV-1 immunopathogenesis in humanized mouse models. *Cellular & Molecular Immunology*.

[6] Zhen A., Rezek V., Youn C. (2017). Targeting type I interferon-mediated activation restores immune function in chronic HIV infection. *The Journal of Clinical Investigation*.

[7] Veenhuis R. T., Freeman Z. T., Korleski J. (2017). HIV-antibody complexes enhance production of type I interferon by plasmacytoid dendritic cells. *The Journal of Clinical Investigation*.

[8] Vermeire J., Roesch F., Sauter D. (2016). HIV triggers a cGAS-dependent, Vpu- and Vpr-regulated type I interferon response in CD4^+^ T cells. *Cell Reports*.

[9] Berg R. K., Rahbek S. H., Kofod-Olsen E. (2014). T cells detect intracellular DNA but fail to induce type I IFN responses: implications for restriction of HIV replication. *PLoS One*.

[10] Asmuth D. M., Murphy R. L., Rosenkranz S. L. (2010). Safety, tolerability, and mechanisms of antiretroviral activity of pegylated interferon alfa-2a in HIV-1-monoinfected participants: a phase II clinical trial. *The Journal of Infectious Diseases*.

[11] Hubbard J. J., Greenwell-Wild T., Barrett L. (2012). Host gene expression changes correlating with anti-HIV-1 effects in human subjects after treatment with peginterferon alfa-2a. *The Journal of Infectious Diseases*.

[12] Azzoni L., Foulkes A. S., Papasavvas E. (2013). Pegylated interferon alfa-2a monotherapy results in suppression of HIV type 1 replication and decreased cell-associated HIV DNA integration. *The Journal of Infectious Diseases*.

[13] Lavender K. J., Gibbert K., Peterson K. E. (2016). Interferon alpha subtype-specific suppression of HIV-1 infection *in vivo*. *Journal of Virology*.

[14] Gargan S., Ahmed S., Mahony R. (2018). HIV-1 promotes the degradation of components of the type 1 IFN JAK/STAT pathway and blocks anti-viral ISG induction. *eBioMedicine*.

[15] Nguyen N. V., Tran J. T., Sanchez D. J. (2018). HIV blocks type I IFN signaling through disruption of STAT1 phosphorylation. *Innate Immunity*.

[16] Swaminathan S., Sui H., Adelsberger J. W. (2014). HIV-1 treated patients with undetectable viral loads have lower levels of innate immune responses via cytosolic DNA sensing systems compared with healthy uninfected controls. *Journal of AIDS & Clinical Research*.

[17] Harman A. N., Nasr N., Feetham A. (2015). HIV blocks interferon induction in human dendritic cells and macrophages by dysregulation of TBK1. *Journal of Virology*.

[18] Kmiec D., Iyer S. S., Sturzel C. M., Sauter D., Hahn B. H., Kirchhoff F. (2016). Vpu-mediated counteraction of tetherin is a major determinant of HIV-1 interferon resistance. *MBio*.

[19] Lo C. C., Schwartz J. A., Johnson D. J. (2012). HIV delays IFN-*α* production from human plasmacytoid dendritic cells and is associated with SYK phosphorylation. *PLoS One*.

[20] Martinelli E., Cicala C., van Ryk D. (2007). HIV-1 gp120 inhibits TLR9-mediated activation and IFN-*α* secretion in plasmacytoid dendritic cells. *Proceedings of the National Academy of Sciences of the United States of America*.

[21] Wie S. H., du P., Luong T. Q. (2013). HIV downregulates interferon-stimulated genes in primary macrophages. *Journal of Interferon & Cytokine Research*.

[22] Yan N., Regalado-Magdos A. D., Stiggelbout B., Lee-Kirsch M. A., Lieberman J. (2010). The cytosolic exonuclease TREX1 inhibits the innate immune response to human immunodeficiency virus type 1. *Nature Immunology*.

[23] Gomez A. M., Ouellet M., Tremblay M. J. (2015). HIV-1-triggered release of type I IFN by plasmacytoid dendritic cells induces BAFF production in monocytes. *Journal of Immunology*.

[24] Alais S., Mahieux R., Dutartre H. (2015). Viral source-independent high susceptibility of dendritic cells to human T-cell leukemia virus type 1 infection compared to that of T lymphocytes. *Journal of Virology*.

[25] Jones K. S., Petrow-Sadowski C., Huang Y. K., Bertolette D. C., Ruscetti F. W. (2008). Cell-free HTLV-1 infects dendritic cells leading to transmission and transformation of CD4^+^ T cells. *Nature Medicine*.

[26] Jain P., Manuel S. L., Khan Z. K., Ahuja J., Quann K., Wigdahl B. (2009). DC-SIGN mediates cell-free infection and transmission of human T-cell lymphotropic virus type 1 by dendritic cells. *Journal of Virology*.

[27] Rizkallah G., Alais S., Futsch N. (2017). Dendritic cell maturation, but not type I interferon exposure, restricts infection by HTLV-1, and viral transmission to T-cells. *PLoS Pathogens*.

[28] Kinpara S., Hasegawa A., Utsunomiya A. (2009). Stromal cell-mediated suppression of human T-cell leukemia virus type 1 expression in vitro and in vivo by type I interferon. *Journal of Virology*.

[29] Kinpara S., Kijiyama M., Takamori A. (2013). Interferon-*α* (IFN-*α*) suppresses HTLV-1 gene expression and cell cycling, while IFN-*α* combined with zidovudin induces p53 signaling and apoptosis in HTLV-1-infected cells. *Retrovirology*.

[30] Macchi B., Balestrieri E., Frezza C. (2017). Quantification of HTLV-1 reverse transcriptase activity in ATL patients treated with zidovudine and interferon-*α*. *Blood Advances*.

[31] Yuen C.-K., Chan C.-P., Fung S.-Y. (2016). Suppression of type I interferon production by human T-cell leukemia virus type 1 oncoprotein Tax through inhibition of IRF3 phosphorylation. *Journal of Virology*.

[32] Diani E., Avesani F., Bergamo E., Cremonese G., Bertazzoni U., Romanelli M. G. (2015). HTLV-1 Tax protein recruitment into IKK*ε* and TBK1 kinase complexes enhances IFN-I expression. *Virology*.

[33] Narulla M. S., Alsairi A., Charmier L. (2017). Positive and negative regulation of type I interferons by the human T cell leukemia virus antisense protein HBZ. *Journal of Virology*.

[34] Armstrong G. L., Wasley A., Simard E. P., McQuillan G. M., Kuhnert W. L., Alter M. J. (2006). The prevalence of hepatitis C virus infection in the United States, 1999 through 2002. *Annals of Internal Medicine*.

[35] Lau D. T., Kleiner D. E., Ghany M. G., Park Y., Schmid P., Hoofnagle J. H. (1998). 10-year follow-up after interferon-alpha therapy for chronic hepatitis C. *Hepatology*.

[36] Meissner E. G. (2017). Update in HIV-hepatitis C virus coinfection in the direct acting antiviral era. *Current Opinion in Gastroenterology*.

[37] Longnecker R. (2000). Epstein-Barr virus latency: LMP2, a regulator or means for Epstein-Barr virus persistence?. *Advances in Cancer Research*.

[38] Feld J. J., Hoofnagle J. H. (2005). Mechanism of action of interferon and ribavirin in treatment of hepatitis C. *Nature*.

[39] Harding H. P., Calfon M., Urano F., Novoa I., Ron D. (2002). Transcriptional and translational control in the mammalian unfolded protein response. *Annual Review of Cell and Developmental Biology*.

[40] Gale M. J., Korth M. J., Tang N. M. (1997). Evidence that hepatitis C virus resistance to interferon is mediated through repression of the PKR protein kinase by the nonstructural 5A protein. *Virology*.

[41] Gale M., Blakely C. M., Kwieciszewski B. (1998). Control of PKR protein kinase by hepatitis C virus nonstructural 5A protein: molecular mechanisms of kinase regulation. *Molecular and Cellular Biology*.

[42] Sugiyama R., Murayama A., Nitta S. (2018). Interferon sensitivity-determining region of hepatitis C virus influences virus production and interferon signaling. *Oncotarget*.

[43] Khabar K. S. A., al-Zoghaibi F., al-Ahdal M. N. (1997). The alpha chemokine, interleukin 8, inhibits the antiviral action of interferon alpha. *The Journal of Experimental Medicine*.

[44] Polyak S. J., Khabar K. S. A., Paschal D. M. (2001). Hepatitis C virus nonstructural 5A protein induces interleukin-8, leading to partial inhibition of the interferon-induced antiviral response. *Journal of Virology*.

[45] Girard S., Shalhoub P., Lescure P. (2002). An altered cellular response to interferon and up-regulation of interleukin-8 induced by the hepatitis C viral protein NS5A uncovered by microarray analysis. *Virology*.

[46] Taylor D. R., Shi S. T., Romano P. R., Barber G. N., Lai M. M. (1999). Inhibition of the interferon-inducible protein kinase PKR by HCV E2 protein. *Science*.

[47] Randall R. E., Goodbourn S. (2008). Interferons and viruses: an interplay between induction, signalling, antiviral responses and virus countermeasures. *The Journal of General Virology*.

[48] Hosui A., Ohkawa K., Ishida H. (2003). Hepatitis C virus core protein differently regulates the JAK-STAT signaling pathway under interleukin-6 and interferon-gamma stimuli. *The Journal of Biological Chemistry*.

[49] Lin W., Choe W. H., Hiasa Y. (2005). Hepatitis C virus expression suppresses interferon signaling by degrading STAT1. *Gastroenterology*.

[50] Lin W., Kim S. S., Yeung E. (2006). Hepatitis C virus core protein blocks interferon signaling by interaction with the STAT1 SH2 domain. *Journal of Virology*.

[51] He C. L., Liu M., Tan Z. X. (2018). Hepatitis C virus core protein-induced miR-93-5p up-regulation inhibits interferon signaling pathway by targeting IFNAR1. *World Journal of Gastroenterology*.

[52] Mukherjee A., Di Bisceglie A. M., Ray R. B. (2015). Hepatitis C virus-mediated enhancement of microRNA miR-373 impairs the JAK/STAT signaling pathway. *Journal of Virology*.

[53] Campbell S. L., Khosravi-Far R., Rossman K. L., Clark G. J., der C. J. (1998). Increasing complexity of Ras signaling. *Oncogene*.

[54] Zhang Q., Gong R., Qu J. (2012). Activation of the Ras/Raf/MEK pathway facilitates hepatitis C virus replication via attenuation of the interferon-JAK-STAT pathway. *Journal of Virology*.

[55] Kuwashiro T., Iwane S., Jinghe X. (2018). Regulation of interferon signaling and HCV-RNA replication by extracellular matrix. *International Journal of Molecular Medicine*.

[56] De Paoli P., Carbone A. (2016). Kaposi’s sarcoma herpesvirus: twenty years after its discovery. *European Review for Medical and Pharmacological Sciences*.

[57] Jung J., Munz C. (2015). Immune control of oncogenic *γ*-herpesviruses. *Current Opinion in Virology*.

[58] Baresova P., Pitha P. M., Lubyova B. (2013). Distinct roles of Kaposi’s sarcoma-associated herpesvirus-encoded viral interferon regulatory factors in inflammatory response and cancer. *Journal of Virology*.

[59] Wies E., Hahn A. S., Schmidt K. (2009). The Kaposi’s sarcoma-associated herpesvirus-encoded vIRF-3 inhibits cellular IRF-5. *The Journal of Biological Chemistry*.

[60] Esteban M., Garcia M. A., Domingo-Gil E., Arroyo J., Nombela C., Rivas C. (2003). The latency protein LANA2 from Kaposi’s sarcoma-associated herpesvirus inhibits apoptosis induced by dsRNA-activated protein kinase but not RNase L activation. *The Journal of General Virology*.

[61] Fuld S., Cunningham C., Klucher K., Davison A. J., Blackbourn D. J. (2006). Inhibition of interferon signaling by the Kaposi’s sarcoma-associated herpesvirus full-length viral interferon regulatory factor 2 protein. *Journal of Virology*.

[62] Mutocheluh M., Hindle L., Areste C. (2011). Kaposi’s sarcoma-associated herpesvirus viral interferon regulatory factor-2 inhibits type 1 interferon signalling by targeting interferon-stimulated gene factor-3. *The Journal of General Virology*.

[63] Burysek L., Pitha P. M. (2001). Latently expressed human herpesvirus 8-encoded interferon regulatory factor 2 inhibits double-stranded RNA-activated protein kinase. *Journal of Virology*.

[64] Chatterjee M., Osborne J., Bestetti G., Chang Y., Moore P. S. (2002). Viral IL-6-induced cell proliferation and immune evasion of interferon activity. *Science*.

[65] Bisson S. A., Page A. L., Ganem D. (2009). A Kaposi’s sarcoma-associated herpesvirus protein that forms inhibitory complexes with type I interferon receptor subunits, Jak and STAT proteins, and blocks interferon-mediated signal transduction. *Journal of Virology*.

[66] Cai X., Lu S., Zhang Z., Gonzalez C. M., Damania B., Cullen B. R. (2005). Kaposi’s sarcoma-associated herpesvirus expresses an array of viral microRNAs in latently infected cells. *Proceedings of the National Academy of Sciences of the United States of America*.

[67] Ramalingam D., Ziegelbauer J. M. (2017). Viral microRNAs target a gene network, inhibit STAT activation, and suppress interferon responses. *Scientific Reports*.

[68] Jain N., Zhang T., Kee W. H., Li W., Cao X. (1999). Protein kinase C delta associates with and phosphorylates Stat3 in an interleukin-6-dependent manner. *The Journal of Biological Chemistry*.

[69] Abend J. R., Ramalingam D., Kieffer-Kwon P., Uldrick T. S., Yarchoan R., Ziegelbauer J. M. (2012). Kaposi’s sarcoma-associated herpesvirus microRNAs target IRAK1 and MYD88, two components of the Toll-like receptor/interleukin-1R signaling cascade, to reduce inflammatory-cytokine expression. *Journal of Virology*.

[70] Doebele C., Bonauer A., Fischer A. (2010). Members of the microRNA-17-92 cluster exhibit a cell-intrinsic antiangiogenic function in endothelial cells. *Blood*.

[71] Rickinson A. B., Long H. M., Palendira U., Munz C., Hislop A. D. (2014). Cellular immune controls over Epstein-Barr virus infection: new lessons from the clinic and the laboratory. *Trends in Immunology*.

[72] Kaye K. M., Izumi K. M., Kieff E. (1993). Epstein-Barr virus latent membrane protein 1 is essential for B-lymphocyte growth transformation. *Proceedings of the National Academy of Sciences of the United States of America*.

[73] Ning S. (2011). Innate immune modulation in EBV infection. *Herpesviridae*.

[74] Geiger T. R., Martin J. M. (2006). The Epstein-Barr virus-encoded LMP-1 oncoprotein negatively affects Tyk2 phosphorylation and interferon signaling in human B cells. *Journal of Virology*.

[75] Richardson C., Fielding C., Rowe M., Brennan P. (2003). Epstein-Barr virus regulates STAT1 through latent membrane protein 1. *Journal of Virology*.

[76] Xu D., Brumm K., Zhang L. (2006). The latent membrane protein 1 of Epstein-Barr virus (EBV) primes EBV latency cells for type I interferon production. *The Journal of Biological Chemistry*.

[77] Mancao C., Hammerschmidt W. (2007). Epstein-Barr virus latent membrane protein 2A is a B-cell receptor mimic and essential for B-cell survival. *Blood*.

[78] Rovedo M., Longnecker R. (2007). Epstein-Barr virus latent membrane protein 2B (LMP2B) modulates LMP2A activity. *Journal of Virology*.

[79] Shah K. M., Stewart S. E., Wei W. (2009). The EBV-encoded latent membrane proteins, LMP2A and LMP2B, limit the actions of interferon by targeting interferon receptors for degradation. *Oncogene*.

[80] Wu L., Fossum E., Joo C. H. (2009). Epstein-Barr virus LF2: an antagonist to type I interferon. *Journal of Virology*.

[81] Middeldorp J. M., Brink A. A., van den Brule A. J., Meijer C. J. (2003). Pathogenic roles for Epstein-Barr virus (EBV) gene products in EBV-associated proliferative disorders. *Critical Reviews in Oncology/Hematology*.

[82] Amoroso R., Fitzsimmons L., Thomas W. A., Kelly G. L., Rowe M., Bell A. I. (2011). Quantitative studies of Epstein-Barr virus-encoded microRNAs provide novel insights into their regulation. *Journal of Virology*.

[83] Hooykaas M. J. G., van Gent M., Soppe J. A. (2017). EBV microRNA BART16 suppresses type I IFN signaling. *Journal of Immunology*.

[84] Yoshimura A., Naka T., Kubo M. (2007). SOCS proteins, cytokine signalling and immune regulation. *Nature Reviews. Immunology*.

[85] Michaud F., Coulombe F., Gaudreault E., Paquet-Bouchard C., Rola-Pleszczynski M., Gosselin J. (2010). Epstein-Barr virus interferes with the amplification of IFNalpha secretion by activating suppressor of cytokine signaling 3 in primary human monocytes. *PLoS One*.

